# Spectrum of candidate gene mutations associated with Indian familial oculocutaneous and ocular albinism

**Published:** 2010-08-09

**Authors:** Kathirvel Renugadevi, Asim Kumar Sil, Vijayalakshmi Perumalsamy, Periasamy Sundaresan

**Affiliations:** 1 Department of Genetics, Dr. G.Venkataswamy Eye Research Institute, Aravind Medical Research Foundation, Aravind Eye Hospital, Madurai, Tamil Nadu, India; 2 Netra Niramay Niketan, Vivekananda Mission Asram, Vivenknagar, Chaitanyapur, West Bengal, India; 3 Paediatric Clinic, Aravind Eye Care System, Aravind Eye Hospital, Madurai, Tamil Nadu, India

## Abstract

**Purpose:**

Albinism is a group of genetic disorders, showing a broad spectrum of different phenotypes. The purpose of this study was to screen known candidate genes for oculocutaneous albinism (OCA) and ocular albinism (OA) mutations in Indian patients.

**Methods:**

Blood samples were collected from 23 probands and 13 affected family members from 23 genetically unrelated Indian families (22 diagnosed as OCA and 1 diagnosed as OA) and analyzed by bidirectional DNA sequencing of the classic OCA genes— tyrosinase (*TYR*, or oculocutaneous albinism IA), pink eyed dilution (*P*; or oculocutaneous albinism II (*OCA2*])*,* tyrosinase-related protein 1 (*TYRP1*)*,* solute carrier family 45, member 2 (*SLC45A2*; or membrane-associated transporter protein [*MATP*])*—*and the OA1 gene, G protein-coupled receptor 143 (*GPR143*).

**Results:**

Three missense mutations, c. 715 C>T (R239W), c. 896 G>A (R299H), c.1255 G>A (G419R), and one termination c. 832 C>T (R278X), were identified in *TYR*, as well as one novel mutation, c.1453 G>A (G485R) in *P*. One novel single nucleotide polymorphism (SNP) was identified in both *TYR* and *P;* few reported SNPs were identified. The G>A base substitution caused relatively conservative amino acid changes, which altered glycine to arginine residues within the topological domain. The novel *OCA2* mutation was not present in 100 control samples. This study identified two probands carrying mutations alone, 16 probands carrying SNPs alone, 4 probands carrying both mutations and SNPs and only one proband carrying neither mutations nor SNPs.

**Conclusions:**

Although sequence analysis was performed with all five candidate genes, only four (17.39%) of the 23 probands showed mutations in *TYR* and 2 probands (8.69%) showed an unreported novel mutation in *P*. Genetic counseling for phenotypical diagnosis and genetic mutation screening of these genes will help to minimize the incidence of OCA and OA in future generations.

## Introduction

Albinism is an autosomal recessive inherited condition present at birth. The phenotype ranges from a complete lack of pigmentation in the skin, hair and iris, called oculocutaneous albinism (OCA), or a lack of pigmentation in the iris alone, termed ocular albinism (OA). Several independent defects can cause albinism including a complete lack of melanocytes or few pigment cells, interference in the migration of the cells to their proper location during embryo development, and failure of the cells to produce melanin due to a lack of tyrosinase or abnormalities within the cells. Albinism is associated with severe photosensitivity to ultraviolet radiation and characteristic abnormalities in the visual system include reduced vision, nystagmus, macular/foveal hypoplasia, misrouting of optic fibers at the chiasm, and greatly decreased visual acuity.

Melanin, a dark pigment, plays an important role in the eyes and brain; it occurs in two forms-black-brown known as eumelanin and yellow-red known as pheomelanin. Both forms are dependent on the activity of the tyrosinase (*TYR*) gene (mutations in *TYR* are responsible for OCA1; OMIM 203100). This gene, which is a copper-containing enzyme located on chromosome 11q14.3 [1], is expressed in melanocytes and controls the major steps in pigment production.

Mutations in the pink eyed dilution gene (*P*; or oculocutaneous albinism II [*OCA2*]) gene (OMIM 203200) cause the OCA type 2 phenotype [2]. The OCA2 protein is important for normal biogenesis of melanosomes [3,4] and normal processing and transport of melanosomal proteins such as *TYR* and tyrosinase-related protein 1 (*TYRP1*) [5,6]. It is thought to control the melanosomal pH value, thus, regulating TYR activity and melanosomal distribution [7-9].

OCA type 3 (OMIM 203290) is caused by mutations in *TYRP1*, which is located on chromosome 9p23 [10]. The protein, which is encoded by the brown locus, exhibits more than 50% sequence identity with tyrosinase [11] and shows some tyrosine hydroxylase activity; it may specifically act as 5,6-dihydroxyindole-2-carboxylic acid (DHICA) oxidase [12,13].

Human solute carrier family 45, member 2 (*SLC45A2*/membrane-associated transporter protein [*MATP*]) (OMIM 606574)—a novel malignant melanoma-associated gene mapping to chromosomal 5p13.3 located in the melanosomal membrane— probably functions as a membrane transporter directing melanosomal protein traffic and other substances to melanosomes [14,15].

Ocular albinism is caused by mutations in the ocular albinism I (*OA1*; or G protein-coupled receptor 143 [*GPR143*]) gene (OMIM 300500). The protein product is thought to be a melanosomal transmembrane protein [16]. *OA1* is expressed in eye and epidermal melanocytes.

We present a molecular analysis of *TYR, P, TYRP1, MATP* and *GPR143*, well known candidate genes for OCA and OA1 types, in 23 (OCA-22 and OA-1) South Indian families (36 affected individuals with a positive history of albinism).

## Methods

### The study group

All participants (16 consanguineous and 7 non-consanguineous familial cases) were referred for molecular analysis by investigators from the Paediatric Clinic, Aravind Eye Hospital, Madurai, Tamil Nadu and Netra Niramay Niketan, Vivekananda Mission Asram, Chaitanyapur, West Bengal, India. The study adhered to the Declaration of Helsinki criteria and was approved by the institutional review board; an informed consent form was obtained from each patient following an explanation of the nature of the study. All the patients were diagnosed using the ophthalmologic examinations detailed in Table 1. Data were also obtained on other ocular conditions such as cataracts, glaucoma, and retinal disease. The controls were selected from 100 healthy individuals with no family history of ocular abnormalities.

**Table 1:** Clinical details of Probands who participated in this study.

		**Ocular region**								**Cutaneous region**		
**Patient ID**	**Age/ Sex**	**Visual Acuity**		**Photo Phobia**	**Iris Pigmentation**	**Type of Refraction Error**	**Fundus**	**Foveal Hypoplasia**	**Nys**	**Hair color**	**Skin color**	**Con**
		**Right Eye**	**Left Eye**									
5–1	7/M	NA	NA	Yes	Hypopigmented	NA	Albinotic	Foveal Hypoplasia	+	brown	white	Con
11–1	24/M	NA	NA	Yes	Blue with gray	NA	Albinotic	Foveal Hypoplasia	-	Reddish Brown	Milky White	Con
14–1	12/M	NA	NA	Yes	Hypopigmented	NA	Albinotic	Foveal Hypoplasia	-	brown	white	Con
16–1	19/F	NA	NA	Yes	Hypopigmented	NA	Albinotic	Foveal Hypoplasia	-	Golden white	Milky white	Con
17–1	6/F	2/60	2/60	Yes	Hypopigmented	Compound Myopic Astigmatism	Albinotic	Macular Hypoplasia	+	Golden white	white	Con
21–1	9/F	2/60	2/60	Yes	Hypopigmented	Simple Myopic Astigmatism	Albinotic	Foveal Hypoplasia	+	brown	white	Con
24–1	15/M	NA	NA	Yes	Hypopigmented	NA	Albinotic	Foveal Hypoplasia	-	white	white	Non Con
25–1	31/M	NA	NA	Yes	Hypopigmented	NA	Albinotic	Foveal Hypoplasia	-	Mild silver brown	white	Non Con
32–1	14/M	NA	NA	Yes	Hypopigmented	NA	Albinotic	Foveal Hypoplasia	-	Golden white	white	Non Con
35–1	9 Months/F	UP	UP	Yes	Hypopigmented	Compound Hypermetropic Astigmatism	Albinotic	Foveal Hypoplasia	+	brown	white	Con
39–1	6/M	6/36	4/60	Yes	Normally Pigmented	Compound Hypermetropic Astigmatism	Albinotic	Foveal Hypoplasia	- (CC)	brown	white	Con
40–1	5/M	NA	NA	Yes	Hypopigmented	NA	Albinotic	Foveal Hypoplasia	-	brown	white	Non Con
41–1	3.5/M	NA	NA	Yes	Hypopigmented	NA	Albinotic	Foveal Hypoplasia	-	brown	white	Con
42–1	13/M	6/24	6/18	Yes	Hypopigmented	Simple Hypermetropic Astigmatism	Albinotic	Foveal Hypoplasia	+	brown	white	Con
44–1	9/M	5/60	5/60	Yes	Hypopigmented	Simple Hypermetropic Astigmatism	Albinotic	Foveal Hypoplasia	+	brown	white	Con
46–1	11/M	6/60	6/36	Yes	Hypopigmented	Compound & Simple Hypermetropic Astigmatism	Albinotic	Foveal Hypoplasia	+	Golden white	Milky white	Con
49–1	24/M	NA	NA	No	Hypopigmented	NA	Albinotic	Foveal Hypoplasia	-	Red with brown	white	Con
50–1	6 Months/F	UP	UP	Yes	Hypopigmented	UP	Albinotic	Foveal aplasia	+	Normal	Normal white	Con
52–1	16/F	3/60	3/60	Yes	Hypopigmented	Simple myopic Astigmatism	Albinotic	Foveal aplasia	+	Golden white	white	Con
54–1	2.5/F	UP	UP	Yes	Hypopigmented	Compound Hypermetropic Astigmatism	Albinotic	Foveal Hypoplasia & Aplasia	+	brown	white	Con
55–1	1.5/M	4/60	4/60	Yes	Hypopigmented	Simple Hypermetropia	Albinotic	Foveal Hypoplasia	+	white	white	NC
58–1	9/M	5/60	5/60	Yes	Hypopigmented	Compound Hypermetropic & Simple Myopic Astigmatism	Albinotic	Foveal Hypoplasia	+	brown	white	Con
62–1	22/F	6/60	6/60	Yes	Hypopigmented	Compound Hypermetropic Astigmatism	Albinotic	Foveal aplasia	+	brown	white	NC

In the table, M-Male; F-Female; CC-Congenital Cataract; Nys-Nystagmus; Con-Consanguineous; NC-Non Consanguineous; UP-Un Predictable (because two patients were 6 and 9 months old); NA-Not Available (Since the patients clinical details was not retrievable because they lost their follow up).

### Preparation of genomic DNA

Approximately 5 ml of blood was collected from each proband and members of their family. Genomic DNA was prepared from peripheral blood leukocytes using the salting-out method [17] and dissolved in TE buffer (1 M Tris-pH 8.0; 0.5 M EDTA-pH 8.0). The DNA was quantified using a Nano spectrophotometer (NanoDrop Technologies, Inc.Wilmington, DL) and subjected to mutation screening analysis.

### PCR amplification of genomic DNA

The genomic DNA was amplified using PCR (MJ Research-PTC-200; Peltier Thermal cycles, Taunton, MA; Eppendorf Mastercycler, Westbury, NY) containing 50 ng of genomic DNA, 0.2 mM concentration of each primer, 200 mM dNTPS (Medox, Biotech PVT.LTD, Chennai, India), 10 mM Tris-HCL (pH 8.3), 50 mM KCL, 1.5 mM Mgcl_2_ and 0.2 U of Taq DNA polymerase (Sigma Aldrich, St. Louis, MO) in 20 ul volume reaction mix. The primer sequences for the intron-exon boundaries and exonic regions were selected for the *TYR* [18,19], *P* [20], *TYRP1* [21], *MATP* [22], and *GPR143* [23] genes. Primer sequences are presented in Table 2. For all amplicons, the genomic DNA was denatured at 94 °C for 5 min followed by 28 cycles of denaturation at 94 °C for 30 s; the annealing temperature differed according to the Tm value of each primer set, extension at 72 °C for 45 s, and final extension at 72 °C for 10 min.

**Table 2:** PCR primer sequences for *TYR*, *P, TYRP1, MATP*, and *GPR143*.

**PCR primers**	**PCR primer sequence (5’-3’)**	**Melting temperature (°C)**	**PCR product size (bp)**
**Tyrosinase (*Tyr*) gene [****18****,****19****]**			
Exon 1-1	F-CAAACTGAAATTCAATAACATATAAG	63	678
	R-GTGGACAGCATTCCTTCTCC		
Exon 1-2	F-TTCAGAGGATGAAAGCTTAAGATAAA	62	521
	R-CGTCTCTCTGTGCAGTTTGG		
Exon 1-3	F-CTGGCCATTTCCCTAGAGC	59	605
	R-CCACCGCAACAAGAAGAGTC		
Exon 1-4	F-CATCTTCGATTTGAGTGCCC	60	514
	R-CCCTGCCTGAAGAAGTGATT		
Exon 1-5	F-CACCCATGTTTAACGACATCA	59	225
	R-GCCAGTCCCAATATGGAATA		
Exon 1-6	F-GACTCTTCTTGTTGCGGTGG	60	252
	R-CCCTGCCTGAAGAAGTGATT		
Exon 2	F-CCAACATTTCTGCCTTCTCC	60	442
	R-TCAGCTAGGGTCATTGTCGAT		
Exon 3	F-AGTTATAAATCAAATGGGATAATC A	60	296
	R-ACATTTGATAGGCACCCTCT		
Exon 4	F-CTGTTTCCAATTTAGTTTTATAC	55	790
	R-TACAAAATGGCCTATGTTAAGC		
Exon 5	F-TGTCTACTCCAAAGGACTGT	59	924
	R-GGCACTTAGCTGGATGTGTT		
**Pink eyed dilution (*P*) gene [****20****]**			
Exon 1	F-GAGTTCTTACTTCGA	52	172
	R-TAAACCCTCCCTGCCTGTTC		
Exon 2	F-GGTGCAAACGTTAGTCTCAG	60	359
	R-CCAATCTGTGTGAAGTCCAC		
Exon 3	F-CTGGGAACACATACATTATT	56	209
	R-GTGCAATGCTCAGAAACTCT		
Exon 4	F-AAGCTTGCTTTGTAGCCATT	65	300
	R-CGATGTGCGGCCACCGCTGC		
Exon 5	F-GAAAAGTGTCTGAGTCTGGC	62	177
	R-GTCCCGAAGGTGCCTGGGTT		
Exon 6	F-ATTTATACCTTACTGCTCTC	58	183
	R-TTTCAGATCTCAGCCAGGCG		
Exon 7	F-GGACATGGGGTTTCTCCTGT	59	264
	R-TGAGATGAAATGAGATTTCAC		
Exon 8	F-AGATCCCAGATGGTGTCTCA	59	213
	R-AGGTCAGACTCCTTTAAACG		
Exon 9	F-AGAGGGAGGTCCCCTAACTG	63	272
	R-ATCTCAAGCCTCCCTGACTG		
Exon 10	F-CTTTCGTGTGTGCTAACTCC	57	195
	R-ACATCTTTGAGCTGACATCC		
Exon 11	F-GCAGCGCTTCATTAGGCTCA	64	201
	R-GGCCAGAGAAGGCCCGGTTA		
Exon 12	F-GTCGTTTTAATATGGTGGCC	56	256
	R-CCTGCAGAAGCAACCTTTA		
Exon 13	F-GCCTCTGTTCTACGAGCCTG	63	231
	R-TGCCAGAACCTGGCCGCAA		
Exon 14	F-TTCACGATGTGTATAGTGGG	59	239
	R-AAGTGGAGGTGTGCGTTTAC		
Exon 15	F-GATTACAGGCGTGAGCCACC	61	293
	R-ACCCATCAACAGATACTTCC		
Exon 16	F-GAGGGTGTTGCTGATATCTG	60	260
	R-GAATGTTCTGCTGCACACCA		
Exon 17	F-AGGCTCCAAGTCACAGACCG	62	219
	R-CTTCTTGGAGAAGTGAATCAG		
Exon 18	F-AGTTGCGTAGGTTATGACAC	58	235
	R-CCCATCCAGAATGTGACAAA		
Exon 19	F-GTTATGTATTTGCAGCCCCT	58	197
	R-AATCCACCAAATACAATTGA		
Exon 20	F-GAATCGGTGTGTTAACAGTG	59	266
	R-GTAGGCTTTCTTCATTCACC		
Exon 21	F-GCCTACCTTATGTTCACGTC	58	212
	R-AATCAAAGAACAGTGGCTGG		
Exon 22	F-TGGTGGGTCTGACCCTAAGT	59	219
	R-AGGCTATGTCCAGGCTAAAG		
Exon 23	F-ACAGTATGGCAGCTTCTCTG	59	229
	R-ACTAACTGTTGCTTTGGGCT		
Exon 24	F-GAGAACAGAAGCTTACCACC	55	204
	R-GCTTAGGAACTAGACAGTTTA		
Exon 25	F-CGTATCTCATGAGCTTATCC	58	574
	R-AGCATACAATTTGAATGCTG		
**Tyrosinase-related protein 1 (*TYRP1*) gene [****21****]**			
Exon 1	F-AGAAGTTCATCAGAGACATC	50	189
	R-TCACCATCATTAATTACATT		
Exon 2	F-CGTGCTTCAGTCTTCTCTACA	59	490
	R-GCAAGGACTTATGAACTCATTC		
Exon 3	F-CGCAAGGCAGATGTTTTCATG	59	416
	R-AAGGCATCTTGTCTGTAAAGA		
Exon 4	F-AGACCAAACAGAAATGAATA	47	305
	R-AAATTCTGACTCCAAGCTATC		
Exon 5	F-AAAGAGCGACAATAAGAACTC	50	319
	R-AAAGCCTTCTCAAAGAAACTT		
Exon 6	F-TTGCTATTACCTGGAAAAGTG	51	275
	R-TGCAAAAAGCATATGAAAATG		
Exon 7	F-ATACGTTGTCTTTGGAATAAT	51	252
	R-ATACCGTGATTACTCTACTTG		
Exon 8	F-TGTCCACTTTTTGGTGATAAC	50	323
	R-ATTCAACCAGGTGGTTTTGTG		
**Membrane-associated transporter protein (*MATP*) gene [****22****]**			
Exon 1-1	F-AGGCTCCACGTCAAATCCAG	63	260
	R-GGTCACATACGCTGCCTCCA		
Exon 1-2	F-CAGACTCATCATGCACAGCA	58	252
	R-ATGCCCACGAGCATCATGAC		
Exon 1-3	F-CAGCATTGTGTGGTTCCTCA	58	261
	R-GGTCAAACACATGAACATCCTC		
Exon 2	F-AACGCGGATGATTCTAAAACAGGA	65	280
	R-CTCATTGTCTGGGGAGCTGA		
Exon 3-1	F-GGGAGTGTCTATGCATGAGG	65	324
	R-GATAGAACCATACTCGTACATTCC		
Exon 3-2	F-GCCCCACTTACAGAGGTTGC	63	224
	R-CAACAAAGAGCAAGAATATTTTCCCTTG		
Exon 4	F-AGCTGGCTGAGTTTCTGCAG	62	265
	R-CCTCAACAGGTGTTAATGGAGG		
Exon 5	F-AGAGGTGGAGAAGCAGAGTG	64	236
	R-GAAGACATCCTTAGGAGAGAG		
Exon 6	F-ATGAGGCACTGCCAGCTGTA	64	286
	R-CCCAAGGCAGAGGTTCAATG		
Exon 7	F-GCCCTAAATGACAGTTCCTTG	58	326
	R-TGTGCTTCACTGTCTCTGAG		
**G protein-coupled receptor 143 (*GPR143*) gene [****23****]**			
Exon 1-1	F-GAGCCTGGCTCTACTGCAGGCGCT	64	250
	R-TGCCCAGGCAGAGCGCGTGGAAGG		
Exon 1-2	F-AGCCACGCAGCTCGTGCTGAGCTTCCAGCC	68	250
	R-CCCAGGCGCTGATCAGATTCCAACCCGCG		
Exon 2	F-TCATTTTTCCAAAGCAAGAAGTCAGC	66	293
	R-GCAGGACGTGAGAACCTGCATT		
Exon 3	F-GTCTACCCTGCCGTCTCAAGGATG	66	248
	R-CGCTCAGTGCCATCTCTTATCTTCC		
Exon 4	F-GTTCCAGGCAGGCCTCTGTGC	68	229
	R-GGCTCATGTATTCCCTGCAAGACAAC		
Exon 5	F-TTTCCCTTTTTGTTCTCATCCTCTTA	63	299
	R-AGGACAACATGTGTCACTGTCTGAG		
Exon 6	F-ACCTGCTTCCATTGCCTTCTCTGTC	68	288
	R-CTTCCCTTTGGAACTTCTGGTCACG		
Exon 7	F-GAAATTCTTCTCTGACTCTCCAGCATT	63	278
	R-TGACAGAGTGAGACCTTGTCTCTGA		
Exon 8	F-ATGGTCCCTTCCAAGCGAGTCC	68	492
	R-TCACATGAGAGGTGCTGCTGAACAC		
Exon 9	F-TGAAAAACTCCATGCACTGAATACT	61	597
	R-TGCATAACTGTACATGTATTTATTTTCTTTTG		

### Mutational analysis

The PCR products were purified using the gel elution kit method (Bio Basic Inc., Toronto, Canada). Bi-directional DNA sequencing for known candidate genes of OCA and OA (for exon- intron boundaries and exonic regions) was performed for all the probands and affected family members. Big dye termination chemistry was employed (3130 Genetic Analyzer; Applied Biosystems, Foster City, CA). The sequencing results were compared to the gene sequences of *TYR*, *P*, *TYRP1*, *MATP*, and *GPR143* using Finch TV and Chromas softwares.

The presence of novel mutations that were identified in the probands was examined in 100 unrelated healthy persons with normal phenotypes to exclude the possibility of polymorphisms. All the mutations were reconfirmed by sequencing with the new PCR product. Heterozygous patients were systematically resequenced to ensure that the screening had not overlooked mutations. To compare the impact of these genes on 22 OCA samples and 1 OA sample, OCA and OA known candidate gene screening was performed for all samples.

Among the families selected, each affected individual (proband) was analyzed for sequence changes in the selected candidate genes. If a sequence change was identified that resulted in alterations in the amino acid sequence in the protein encoded by the gene, the rest of the family members were analyzed for co-segregation of the genotype.

Evolutionary conservation of human variant amino acid residues was evaluated using Expasy tools by alignment to pig (*Sus scrofa*), mouse (*Mus musculus*), oryla (*Oryzias latipes*), astfa (*Astyanax fasciatus*), and nemve (*Nematostella vectensis*).

### Restriction enzyme cleavage analysis

The presence or absence of a restriction site was used to detect the co-segregation of sequence variations in family members and the control population. Free online software (Insilico) was used to identify the appropriate restriction enzyme. Restriction digestion of PCR products was performed as per the recommendations of the respective manufacturers (New England BioLabs, Beverly, MA). For 1 ug of DNA, 10 units of restriction enzyme was used and incubated at the recommended temperature overnight and analyzed using 2% pre-stained agarose gel.

## Results

### Clinical criteria

A summary of the available clinical data are given in Table 1. Few families alone lack the clinical details that include visual acuity and type of refraction error. Most patients exhibited hypopigmented iris, nystagmus, photophobia, refractive error with combined myopic or hypermetropic astigmatism, and albinotic fundus with foveal hypoplasia.

### Identification of mutations

Bi-directional DNA sequence analysis revealed four mutations (R239W, R278X, R299H, and G419R) in OCA type I (*TYR*) and one novel mutation (G485R) in OCA type II (*P*) among 23 probands and 13 affected individuals from the 23 familial cases (recruited from 80 families). As shown in Table 3, four probands showed mutations in *TYR* (17.39%), and two probands expressed the same novel mutation in *OCA2* (8.69%).

**Table 3:** Mutations and polymorphisms in *TYR*, *P, TYRP1, MATP*, and *GPR143*.

				**Mutations**		
**Gene**	**Patient ID**	**Exon**	**Intron**	**Alteration in c.DNA**	**Alteration in protein**	**SNPs**
*TYR*	55–1	1		c. 715 C>T	R239W	I222V*±
	35–1	2		c. 832 C>T	R278X	
	40–1	2		c. 896 G>A	R299H	
	24–1	4		c.1255 G>A	G419R	
	49–1	1				I222V*±
	32–1	1 & 4				I222V*±& R402Q ±
*P*	11–1, 52–1	14		c.1453 G>A	G485R#	
	17–1, 21–1, 24–1, 35–1, 46–1, 49–1, 54–1, 55–1, 58–1 (H)		20			IVSXX+4 A/G*
*TYRP1*	25–1	2				Arg87Arg
*MATP*	16–1, 44–1 (h)					
	5–1, 14–1, 17–1, 21–1, 35–1, 41–1, 52–1, 58–1, 62–1 (H)	4				Thr329Thr
	42–1 (h)	5				Leu374Phe
	32–1 (H)	7				rs45552240
*GPR143*	50–1 (h)					
	24–1, 46–1, 49–1, 58–1 (h)					
	54–1, 21–1, 32–1, 52–1 (H)		6			IVSV1+10C/G

In the table, # indicates a novel homozygous mutation (PMID: 19309806); the asterisk indicates a novel polymorphism; (H) indicates Heterozygous; and (h) indicates - Homozygous. Among the 23 Albinism families, the probands from 22 OCA families shows either mutation or polymorphism except the proband from family 39–1, In *GPR143*, one SNP was observed on the OA proband alone. Among the candidate gene analysis, a novel mutant was observed on *OCA2* that was absent in 100 alleles of ethnically matched controls and could represent potential amino acid change in the gene.

In family 55, 11 members (two affected and nine unaffected) underwent bi-directional DNA sequencing analysis. The analysis revealed that the proband (55–1) was homozygous for a c.715C>T mutation. This mutation was also identified as homozygous in the proband’s maternal grandmother who had albinism. Both parents showing the normal phenotype were heterozygous for the mutation. The genotype status was further confirmed by SsiI restriction enzyme digestion of exon 2 PCR product amplified from the DNA of the proband’s unaffected parents. Both parents were confirmed to be heterozygous for the c.715C>T mutation. The maternal grandfather and grandmother were heterozygous and mutant homozygous, respectively. The remaining unaffected members of the family exhibited a wild type of the mutation. The wild type sample following SsiI restriction enzyme digestion revealed three (166 bp, 72 bp, and 14 bp) fragments, whereas the mutant sample showed only two fragments (238 bp and 14 bp), as a result of the loss of one SsiI digestion site (data not shown).

In family 35, base changes in the c.832C>T mutation were observed in both alleles, that codon results (R278X) termination of protein synthesis in proband (35–1). Bi-directional sequence analysis of exon 2 PCR-amplified DNA of the proband’s unaffected mother and maternal grandmother revealed the carrier status of (R278X) stop codon.

In family 40, a c.896G>A transition was identified on both alleles in the proband (40–1), and the proband’s unaffected father was heterozygous for the stop codon with carrier genotype.

Similarly in family 24, the c.1255G>A transition was identified in exon 4 of the proband (24–1) as homozygous; no other family members were willing to undergo further analysis.

**Figure 1:**
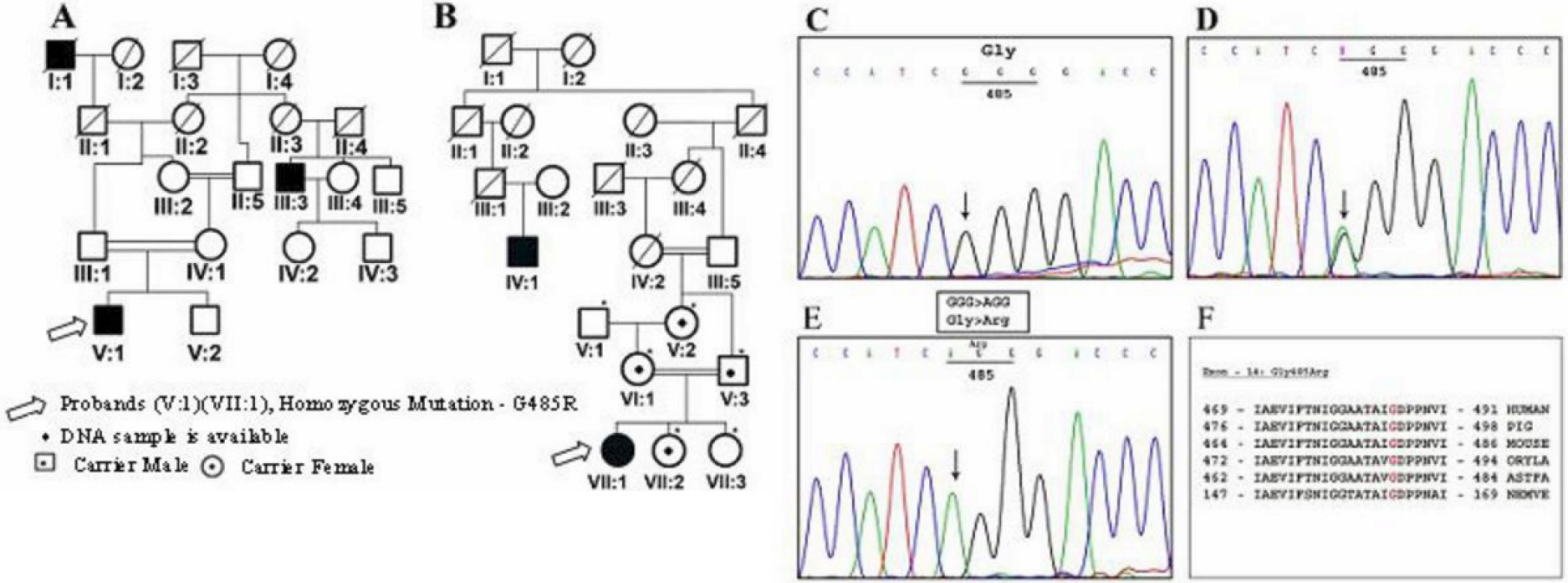
Pedigree and chromatogram of novel mutation Gly485Arg. **A**: Pedigree obtained from family 11. **B**: Pedigree obtained from family 52. **C**: Normal genotype from control samples. **D**: Heterozygous genotype from unaffected father (V:3), mother (VI:1), maternal grandmother (V:2), and first younger sister (VII:2). **E**: Mutant genotype from both Probands (V:1) and (VII:1) from family 11 and 52. **F**: Evolutionary conservation of Gly485Arg among the other related mammalian species. The amino acid residue glycine in the 485th position shown in red are evolutionary conserved.

Interestingly, the probands from families 11 (Figure 1A; V:1) and 52 (Figure 1B; VII:1) were found to be homozygous for the novel mutation c.1453G>A identified in exon 14 of the *P* gene, responsible for OCA type II. One hundred individuals with normal phenotypes were screened for this novel mutation to exclude the possibility of polymorphisms. The degree of consanguinity in both families was high, with both parents marrying first-degree relatives. The family members of pedigree 11 were not willing to participate in this study so no further analysis could be undertaken. We, therefore, have no proof of G485R inheritance in family 11 alone. However, in family 52 the diseased allele was heterozygous in the proband’s unaffected father, mother, maternal grandmother, and first younger sister.

### Identification of polymorphisms

Although bi-directional DNA sequencing revealed five different polymorphisms in the exon, intron, and 3′UTR region of *TYR, P, TYRP1, MATP*, and *GPR143*, no pathological gene mutations were detected among the selected candidate genes in a few probands. Thus, we report on the polymorphisms we identified. In the study group, two probands (55–1, 49–1) exhibited an I222V polymorphism and one proband (32–1) had I222V and R402Q polymorphisms in *TYR* (13.04%). All three probands exhibited heterozygous patterns for these SNPs in the regions analyzed. Both of the SNPs in *TYR* were not observed in any of the control samples. In *P*, the SNP IVSXX+4 A/G was observed in nine different probands (39.13%) in the heterozygous form; similarly, heterozygosity was observed in three of the 100 controls used in the analysis. In *TYRP1,* proband 25–1 (4.34%) alone showed an R87R polymorphism in one allele; this SNP was not identified in the 100 healthy controls. In *MATP*, 13 different (56.52%) probands exhibited polymorphisms. Eleven probands carried the SNP T329T (two probands were homozygous and nine probands were heterozygous). This SNP was also detected in 16 of the 100 controls. Apart from this polymorphism one proband 42-1 carried the SNP, Leu374Phe, in exon-5 and rs45552240 (SNP) was observed in 32-1 on exon 7. Similarly, nine different (39.13%) probands exhibited IVSV1+10C/G polymorphisms in *GPR143* (eight probands from OCA-24–1; 46–1; 49–1; 58–1; 54–1; 21–1; 32–1; 52–1 and one proband from OA-50–1). Table 3 represents a list of the mutations and polymorphisms.

## Discussion

There is an increased awareness of genetic diseases in all of the races. Identification of mutations in candidate genes will facilitate DNA-based diagnosis of albinism. Molecular analysis of pathological mutations to reveal the origin of mutated alleles in a study population is useful under certain circumstances such as understanding the nature and severity of mutations to deliver genetic counseling. We identified four known mutations in *TYR* and one novel mutation in *P* in 23 unrelated families. The clinical categorization of an individual has become more unique and complex. Clinical references are useful and are sufficiently precise for initial diagnosis and family counseling. Accurate diagnosis of most genetic disorders is only possible with candidate gene analysis. Of the four mutations identified in the patients with albinism, three were missense and one was a nonsense (termination) mutation. These mutations were mainly clustered in exons 1, 2, and 4 (Table 3) of *TYR*. In the entire proband sample, four of 23 (17.39%) individuals were homozygous for a *TYR* mutation; consanguinity was associated with two of these four mutations.

The R239W missense substitution in *TYR* in a patient (55–1) from family 55 with tyrosinase-negative OCA was previously reported in Japanese patients,100%, 6.25%, respectively [24,25],  and Chinese patients, 0.83% [26]. The 4.34% frequency we identified in our study group does not match any of these earlier findings.

In proband 35–1, the exon 2 direct sequence reveals a homozygous change, c.832C>T, that would create a premature stop codon, R278X, resulting in a truncated and completely inactive enzyme lacking one potential copper-binding region. This mutation was previously reported in various ethnic groups Guayanan, 12.5% [27]; Jewish, 2.6% [7]; Japanese, 100%, 12.5%, 22.2%, respectively [8,25,28]; European, 2.5% [26]; Mexican, 0.83% [26]; Indian, 0.83% [26]; Syrian, 0.83% [26]; Eastern Indian, 25%, 100%, 8.3%, respectively [18,29,30]; Indian, 80% [31]; and Chinese, 18.75% [32]. The frequency of the mutation in our study, 4.34%, does not correlate with that reported in the previous studies. The fact that c.832C>T has been reported in different ethnic groups suggests that c.832C>T may represent a mutation hot spot in different populations.

One missense substitution, R299H, was detected in proband 40–1 despite the fact that the child was the offspring of a non-consanguineous marriage. The heterozygous missense mutation was present in the proband’s unaffected father. The R299H substitution has been observed previously in Caucasian, 12.5% [33]; Arab-Christian, 2.6%, 1.6%, 3.3%, respectively [7,26,34]; and Chinese, 18.75% [32] populations. We detected a 4.34% frequency of the R299H mutation, an approximate match to that reported earlier in Arab-Christian populations [34]. The G419R mutation was identified in exon 4 of proband 24–1. This pathological mutation has been previously reported in Indo-Pakistani, 25% [27]; Caucasian, 0.83% [26]; Pakistani, 0.83% [26]; Indian, 20% [31] and South-Indian, 16.6% [30] populations. Once more, the frequency, 4.34%, of the mutation in our study patient showed no correlation with previous findings.

We identified a novel missense mutation, c.1453G >A (G485R) in *P* of two genetically unrelated patients. In family 52, the proband’s parents, maternal grandmother and first younger sister are carriers of the mutation. The finding sheds new light on the *P* gene mutation and highlights the importance of analyzing this gene in Indian patients. Our results also indicate that the frequency of mutations in the OCA2 gene in Indian patients is to some extent comparable to that seen in Caucasian patients [35]. The presence of this mutation in the *OCA*2 gene is the first report in an Indian patient [36].) A novel c.1454G>T (p.G485V) mutation has been recently reported in a Danish population [37] in the same codon.

We observed one heterozygous novel SNP c.664A>G (I222V) in *TYR* in three different patients (32–1, 49–1, 55–1). The SNP rs34878847 (c.665T>C) has been reported in the same codon. In family 32, the proband 32–1 and his affected father were heterozygous for a c.1205G>A transition, (R402Q), in exon 4 of *TYR*. The heterozygosity was confirmed using Hpy188I RFLP analysis, in which mutation results in the loss of one restriction site; digestion reveals 368 bp, 182 bp, 150 bp, 50 bp,and 41 bp products in wild-type samples, whereas in heterozygous individuals it produces 368 bp, 22 3bp, 182 bp, 150 bp, 50 bp, and 41 bp fragments. In both instances, products below 100 bp were not clearly visible in agarose gel electrophoresis (data not shown). R402Q, a common non- pathological polymorphism of the human tyrosinase gene, was previously reported in Caucasians [38]. Here, the substitution of glutamine for arginine at codon 402 results in moderate thermoinstability of the corresponding tyrosinase polypeptide [38].

One novel intronic SNP IVSXX+4 A/G was heterozygous in *P* from nine different probands. The rs34509359 (R87R) SNP has been previously reported in *TYRP1* [39]. We detected the same SNP in patient 25–1 as heterozygous.

In *MATP*, we identified a common synonymous SNP rs2287949, (T329T), in exon 4 as homozygous in two different patients and heterozygous in another nine patients. In the same gene, a non-synonymous SNP rs16891982, (L374F), in exon 5 was homozygous in one patient (Table 3). Both these SNPs have been previously reported in a Turkish population and an East Indian population [40,41]. L374 significantly increases the possibility of having black hair and, thus, may be considered a future marker for the prediction of black hair color [42]. In the MATP coding sequence, one 3′UTR SNP, rs45552240, was heterozygous in patient 32–1.

In *GPR143,* the intronic SNP rs3788938 was heterozygous in four patients and homozygous in five others.

Taken together, our results indicate that six probands harbored five mutations (four reported and one novel in two probands) and that more than half of affected individuals who were tested (29 among 36) exhibited no apparent pathological mutations in the selected candidate genes. However, homozygous and heterozygous polymorphisms were distributed among the selected candidate genes across most of those with the disease, except one patient (39–1) who showed normal iris pigmentation. The prevalence of inherited ophthalmological diseases in our study is associated with the high rate of consanguineous marriage that we observed. Our study contributes to the development of mutation detection methods for OCA and OA in South Indian families. The identification of prevalent or novel alterations in candidate genes will shed new light on studies of expression analysis and may reveal alterations in mutated alleles suitable for further functional studies. Further analyses are needed to provide insight into the structure-function relationships of mutations involved in the candidate genes for albinism.
